# AI‐Driven Recruitment for Alzheimer's Disease Clinical Trials: A Pilot Analysis on the A4 Dataset

**DOI:** 10.1002/alz70856_105402

**Published:** 2026-01-08

**Authors:** Luis R Peraza, Robin Wolz, Richard Joules

**Affiliations:** ^1^ IXICO, London, London, United Kingdom

## Abstract

**Background:**

Efficient recruitment is a challenge in Alzheimer's disease (AD) clinical trials, and deployment of sensitive and specific biomarkers early in the screening funnel can help screen out non‐AD cases. We previously trained an AI model for diagnostic classification of AD, frontotemporal dementia (FTD), and healthy control (HC) using T1‐weighted MR images from 2,520 AD, 182 FTD, and 1,190 HC participants from the ADNI, OASIS, EPAD, FTLDNI, BrainLat datasets (Wolz et al., ADPD 2025). Here, we evaluate the model's ability to improve recruitment efficiency as a pre‐screening tool on the A4 Study dataset.

**Methods:**

Brain volumetry was computed from a random subset of the A4 dataset (*N* = 342, age=70(±4.7), MMSE=29(±1.2), and SUVR=1.14(±0.18)) and fed to the pretrained model to obtain clinical group predictions. Suitable candidates were defined as those with SUVR>=1.1 (*n* = 166). We assessed screen failure rate (SFR) and sensitivity of the following pre‐screening methods: MMSE<=29, normalised hippocampal volume (HVn, <75 percentile threshold), AI‐based class probability for AD (>25 percentile) and FTD (<75 percentile), as well as the interactions MMSE×HVn, MMSE×AD‐class probability, and MMSE×FTD‐class probability with previously defined thresholds.

**Results:**

Our MRI‐based model classified the test dataset as 68% HC, 19% AD, 4% FTD and 9% unknown. Spearman's correlation between SUVR and HC probabilities was negatively correlated r=‐0.25 (*p*‐value<0.002) after a 1.1 SUVR knot value. FTD probabilities were ‐0.14 (Spearman's, *p*‐value<0.01). No significant correlations between the AD‐class probabilities and SUVR were observed.

Without pre‐screening SFR was 51SFR was 51.5% with 100% sensitivity (*n* = 166 suitable participants). Pre‐screening with MMSE reported a SFR=52.2% with 60.2% sensitivity (*n* = 100) while hippocampal volume showed an SFR=48.4% with 79.5% sensitivity (*n* = 132). The interaction MMSE×HVn showed an SFR=48.2% with 77.7% sensitivity (*n* = 129). The AD‐class probability threshold showed an SFR=49.2% and 78.3% sensitivity (*n* = 130), the FTD‐class probability resulted in 47.0% SFR and 80.1% sensitivity (*n* = 133, *p*‐value=0.002). The MMSE×AD probability interaction showed 48.8% SFR and 74.7% sensitivity (*n* = 124) and the MMSE×FTD interaction showed 49.3% SFR and 89.8% sensitivity (*n* = 149).

**Conclusions:**

A pretrained AI classification model can improve recruitment efficiency and participant sensitivity in new clinical trials outperforming screening using only MMSE. Further research is warranted to study the cost benefits of our proposed approach.

